# Reconfigurable elastomeric graded-index optical elements controlled by light

**DOI:** 10.1038/s41377-018-0005-1

**Published:** 2018-05-23

**Authors:** Angelo Angelini, Federica Pirani, Francesca Frascella, Emiliano Descrovi

**Affiliations:** 1 Department of Applied Science and Technology (DISAT), Politecnico di Torino, C.so Duca degli Abruzzi 24, Torino, IT-10129 Italy; 2 Center for Sustainable Future Technologies@PoliTo, Istituto Italiano di Tecnologia, C.so Trento 21, Torino, IT-10129 Italy

## Abstract

In many optical applications, there is an increasing need for dynamically tunable optical elements that are able to shape the wavefront of light ‘on demand’. In this work, an elastomeric easy-to-fabricate optical element whose transmission functions can be reversibly phase configured by visible light is demonstrated. The light responsivity of proper azopolymers incorporated within an elastomeric matrix is exploited to induce a light-controlled graded refractive index (GRIN) distribution within the bulk compound. The induced refractive index distribution is continuous and conformal to the intensity profile of the illumination at moderate power. A 100 mW doubled-frequency Nd:YAG Gaussian beam focused to a 650 μm waist is shown to induce a maximum relative refractive index change of ~0.4% in the elastomeric matrix, with an approximately parabolic profile. The restoring characteristics of the elastomeric matrix enable full recovery of the initial homogeneous refractive index distribution within a few seconds when the incident laser is switched off. As an exemplary application, the configurable GRIN element is used in a microscope-based imaging system for light control of the effective focal length.

## Adaptive optics: bouncy solution to on-demand focusing

By incorporating photosensitive molecules into a rubbery matrix, researchers have created a lens that can be dynamically re-shaped using visible light. Most tunable lenses rely on special lithographic patterns to manipulate light, but natural systems such as the human eye use variable refractive index changes for adjustable focus. Emiliano Descrovi at the Polytechnic University of Turin in Italy and colleagues have developed an optical element flexible enough to deliver refractive index gradients through laser beam writing. The team found that azopolymers — compounds that switch orientation and expand under light stimulus — could introduce predictable, parabolic refractive index changes of up to 0.4% inside a polydimethylsiloxane substrate after pinpoint irradiation. Imaging experiments demonstrated the possibility of resolving objects by manipulating laser exposure times to progressively increase the new lens’ focal length.

## Introduction

In recent years, many types of programmable optical elements based on different concepts have been proposed, providing specific functionalities in optical microsystems, such as adjustable focal length^1–4^, optical manipulation^5^, holographic projection^6^, and aberration correction capabilities^7–9^. In this framework, liquid crystals (LC) have been extensively used for wavefront engineering applications^10,11^ because of the ability to tune optical properties by application of an external electric voltage. A recent study has reported the imaging capabilities of an LC-based microlens array wherein the focal length can be electrically varied^12^. However, such systems based on electrically driven birefringence exhibit a few drawbacks such as a limited phase modulation depth due to constraints on the LC cell thickness^13^ and sensitivity to the polarization of light, while polarization-free LC-based tunable lenses can be realized but at the cost of increased complexity for the fabrication process^14^.

In addition to LC-based devices, liquid lenses have also attracted great interest^15^. In a liquid lens, an accurate modification of the transmission function^16^ can be achieved by varying the curvature of the interface between liquids with different refractive indices by means of external mechanical pressure/forces^17^, electrowetting^18^, and dielectrophoresis^19^. As an intriguing alternative, flexible membranes made of stimuli-responsive materials can be utilized as tunable lenses, wherein the focal length is controlled by external physical/chemical stimuli, such as temperature, pH or electromagnetic fields^20,21^. Liquid or membrane-based lenses with adjustable focus capabilities can mimic the operation of the human eye, which remains one of the most high-performance optical systems in nature. However, the eye’s ability to image objects at different distances with almost no aberrations relies both on mechanical actuation and a graded refractive index (GRIN) distribution, which is typically not provided in state-of-the-art active lenses^22^.

In this work, we propose an adaptive GRIN optical element whose transmission function can be reversibly varied by means of laser irradiation, without the need for pre-patterning. More specifically, we demonstrate that the refractive index of a flexible element can be decreased according to the intensity pattern of laser illumination. As a consequence, as a stretchable suspended membrane^8^, the proposed optical element is suitable for control by both optical and mechanical stimuli, wherein the light-controlled GRIN distribution compensates for eventual spherical aberrations. Unlike other pixelated devices, such as optically addressed Spatial Light Modulators^23^, electrical biases are not required here, thus enabling one to fabricate thicker elements wherein larger phase modulations up to 30*π* can be achieved. The GRIN element consists of a cross-linked elastomeric blend (polydimethylsiloxane, PDMS) hosting Poly (Dispersed Red 1 methacrylate) (pDR1M) and a specific azopolymer whose light responsivity has been previously extensively studied^24^. Azopolymers are well known because of their ability to convert optical stimuli into a molecular response, resulting in macroscopic effects, such as light-induced birefringence^25^, directional mass migration^26–28^, volume variations^29^, photohardening^30^ or photosoftening^31^ and mechanical actuation^32^ depending on the different formulations. In a recent study, a cross-linked polymeric compound containing azopolymers were demonstrated to reversibly and isotropically expand under moderate laser radiation sufficient to trigger a photoisomerization process^33^. Here we show that the spatial distribution of the refractive index change associated with such a light-induced volume expansion can be exploited in an optical phase function for wavefront shaping of any other incident radiation whose frequency falls outside the absorption bands of the azo-groups. The cross-linked PDMS hosting the azopolymer limits mass-migration effects, thus limiting irreversible plastic deformations during irradiation^27^. Furthermore, the elastomeric feature of PDMS provides a restoring mechanism to the initial condition, as soon as the optical stimulus is turned off.

**Fig. 1:**
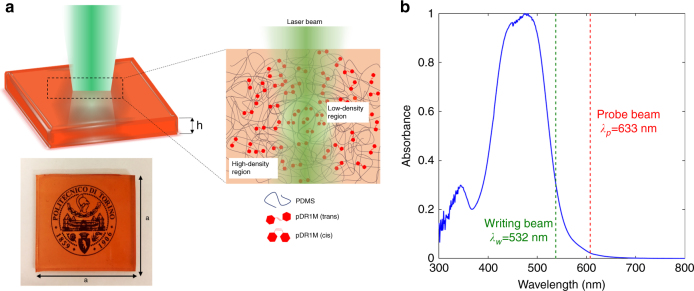
Photoresponsive elastomer. **a** Sketch and actual photograph of the PDMS-pDR1M sample showing the ‘writing’ beam and resulting light-induced density variation effect within the elastomeric matrix. **b** Absorption spectrum of the cross-linked PDMS-pDR1M compound. The spectral positions of the ‘writing’ beam and the ‘probe’ beam are indicated by the green dashed line and the red dashed line, respectively

As sketched in Fig. 1a, a ‘writing’ laser beam with a wavelength of *λ*_*w*_ = 532 nm is used to illuminate the sample and trigger the pDR1M photoisomerization (*trans*-*cis*). As a result, a corresponding decrease in the density and refractive index is produced within the PDMS network. To quantitatively evaluate the variation in the refractive index, an interferometric system based on a Mach–Zehnder configuration is used. Due to sample transparency at wavelengths above 600 nm (Fig. 1b), a well-collimated *λ*_*p*_ = 633 nm ‘probe’ beam superposed onto the ‘writing’ beam is transmitted through the polymeric slab and collected by a long working-distance objective for subsequent phase imaging. A detailed description of the interferometric imaging apparatus is provided in the Experimental Section.

## Materials and methods

### Elastomeric GRIN element fabrication

The device is a mixture of polydimethylsiloxane (PDMS, from Dow Corning, Sylgard 184) and Poly (Dispersed Red 1 methacrylate) azopolymer formulation (pDR1M). The elastomeric compound containing the azopolymer is obtained via a mixing and drying method. The pDR1M is dissolved in toluene (anhydrous, ≥99.8%, Sigma-Aldrich) at a 2 wt.% concentration. The PDMS prepolymer is obtained by mixing the elastomer solution together with the curing agent in a proper ratio (10:1, w/w). The mixture is left in a desiccator until the air bubbles are completely removed. A total of 70 µl of pDR1M is mixed into 5 ml of the PDMS prepolymer solution and vigorously stirred until the azopolymer is completely dissolved in the host material. Mixing is a critical step since azopolymer clots can lead to scattering effects, which should be carefully avoided. For this reason, we employ pDR1M because of its miscibility with PDMS, thus enabling a good optical quality (Supporting Information). The resulting blend solution is left at room temperature to allow the solvent to completely evaporate. Then, the mixture is thermally cured by placing it in an oven at 60 °C for 2 h. Finally, a transparent and flexible PDMS-based material is obtained. The PDMS is molded as a square plate with a side length of 2.5 cm and a thickness of 2.7 mm. The reddish color is due to the pDR1M absorption band, which enables the sample to be light triggered by green light irradiation.

### Optical setup

**Fig. 2:**
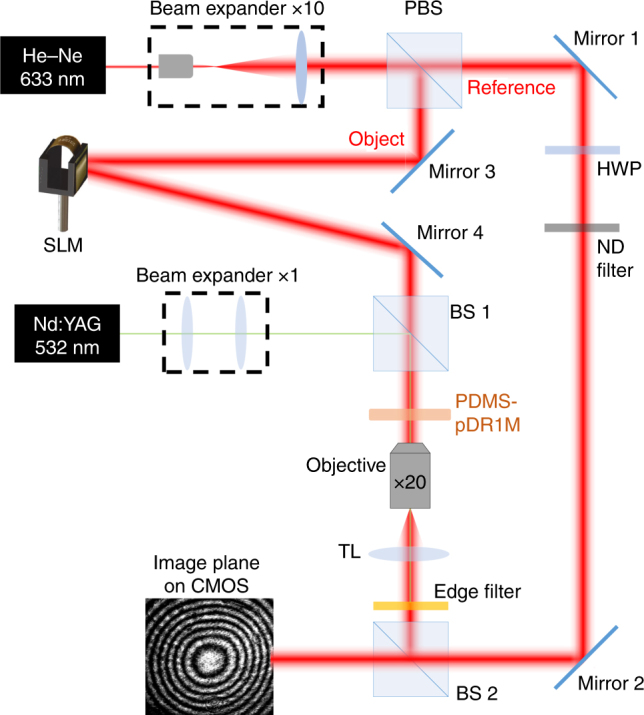
Interferometric imaging system. Schematic view of the interferometric microscope used for the characterization of the optical response of the elastomeric GRIN element

A Mach–Zehnder interferometer is used for quantitative estimation of the light-induced GRIN distribution in the elastomeric compound, as shown in Fig. 2. A He–Ne laser beam (*λ*_*p*_ = 633 nm) is expanded and split into a reference beam and an object beam by a polarizing beam splitter (PBS). The fringe contrast is controlled by adjusting the intensity and polarization of the reference beam using a half-wave plate (HWP) and neutral density (ND) filter. The object beam is diffracted by a phase-only spatial light modulator (SLM—Holoeye Pluto vis-006c), which sequentially generates five *π*/2 phase shifts for phase retrieval^34^. The first-order diffracted beam, referred to as the ‘probe’ beam, is transmitted through the sample and collected by an objective (Mitutoyo Long Working Distance, ×20 0.42 NA). A tube lens (TL) is used to project the image in the object plane of the objective onto a CMOS camera (Thorlabs DCC14p35M), where the reference beam is also superposed after passing through a beam splitter (BS2). Finally, an interferogram is recorded for each phase shift provided by the SLM. Phase maps are obtained by a proper combination of the five interferograms. A phase-unwrapping process based on the Goldstein method is then performed^35^. The imaging system is arranged such that the wavefront incident on the CMOS camera is flat (within the objective field of view) when no sample is placed beneath the objective.

The interferometer is also equipped with a doubled-frequency Nd:YAG laser emitting a TEM_00_ Gaussian beam at a wavelength of *λ*_*w*_ = 532 nm, with a tunable power of up to 200 mW. This ‘writing’ beam is superposed onto the ‘probe’ beam and used to induce refractive index modifications in the PDMS-pDR1M slab. Following a beam expansion stage (Beam expander 1X) and a beam splitter (BS 1), the maximum power incident onto the sample is 100 mW. The fraction of the ‘writing’ beam power transmitted through the sample is then filtered by an edge filter (Semrock RazoEdge MaxLine 532) before reaching the CMOS camera to avoid disturbing the interferogram recording.

## Results and discussion

**Fig. 3:**
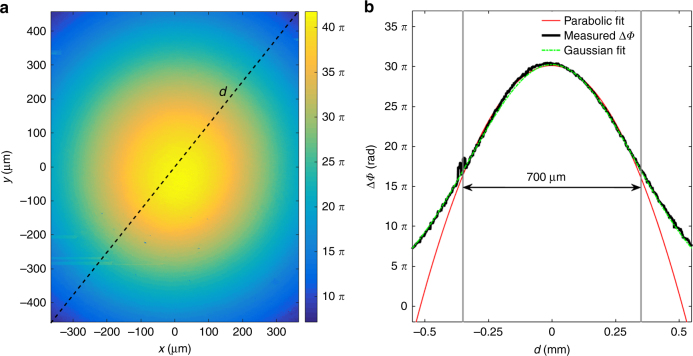
Photoinduced GRIN distribution. **a** Unwrapped phase map of the ‘probe’ beam transmitted through the PDMS-pDR1M slab. **b** Cross-sectional phase profile (baseline subtracted) along a diagonal cut and corresponding parabolic fit within the 700 μm-wide fit interval (red dashed line)

The ‘writing’ irradiation is a linearly polarized Gaussian beam having a beam waist of *w*_0_ ≈ 650 μm that is used to perpendicularly illuminate a 2.7 mm-thick PDMS-pDR1M slab. The collection objective is positioned such that the top surface of the sample is imaged onto the camera. Figure 3a shows the unwrapped phase map retrieved for the ‘probe’ beam transmitted through the slab during irradiation by the ‘writing’ beam (power 100 mW). The unwrapped phase has an axis-symmetric convex profile, meaning that the transmitted ‘probe’ beam is divergent along the forward propagation direction. This effect can be explained by invoking an optical path decrease corresponding to the region irradiated by the ‘writing’ beam, which is in agreement with the expected light-induced mass density decrease described elsewhere^29^. As the unwrapped phase is determined up to a constant, such a baseline value is first determined by means of a Gaussian fitting and then subtracted. Since the imaging system has a limited field of view, the measured phase is greater than zero on the map boundaries.

For a better evaluation of the phase profile, a cross-section along a diagonal line (black dashed line in Fig. 3a) is shown in Fig. 3b. Interestingly, the phase profile is Gaussian, similar to the ‘writing’ beam. However, within a range of ~700 μm centered on the symmetry axis, the phase profile is well fitted by a parabolic function (red dashed line in Fig. 3b), as the first term in the Taylor expansion of a Gaussian is a quadratic power. This suggests that this central region of the light-induced GRIN distribution can actually operate as a concave lens with a negative focal length (paraxial approximation). In addition, the laser-induced refractive index change shows basically no birefringence or optical anisotropy (see the section Polarization Sensitivity in Supporting Information).

For a rational use of this light-induced lens effect, a predictive mathematical model is required. Therefore, a finite element method for ray-tracing (FER) in graded-index media^36^ has been implemented. The model computes ray trajectories within a medium characterized by a given GRIN distribution; therefore, a quantitative estimate of the refractive index distribution within the PDMS-pDR1M volume is essential. Taking the direction (0, 0, *z*) as the optical axis, the GRIN distribution *n*(*x, y, z*) can be expressed conformally to the ‘writing’ beam intensity distribution as follows:

1 $$n\left( {x,y,z} \right) = n_0 - n_{{\rm ind}}\left( {x,y,z} \right)$$

2 $$n_{{\rm ind}}\left( {x,y,z} \right) = n_{{\rm ind}}^{\max } \times \left( {\frac{{w_0}}{{w\left( z \right)}}} \right)^2 \times e^{ - \frac{{2x^2}}{{w\left( z \right)^2}}} \times e^{ - \frac{{2y^2}}{{w\left( z \right)^2}}} \times e^{ - \alpha z}$$

where $n_0 = 1.41$ is the unperturbed refractive index; $n_{{\rm ind}}$ is the light-induced refractive index; $n_{{\rm ind}}^{\max }$ is the maximum value of the light-induced refractive index (corresponding to the maximum intensity of the ‘writing’ beam, at *x* =* y* = *z* = 0); *w*_0_ = 650 μm is the ‘writing’ beam waist; *w*(*z*) is the beam diameter at a distance *z* from the waist position; and *α* is an attenuation coefficient at *λ*_*w*_ = 532 nm. In our model, nonlinearity is not considered; therefore, the intensity distribution of the ‘writing’ beam is assumed to be independent of the light-induced refractive index change in the medium. According to the experimental configuration, the waist position corresponds to the entrance side of the PDMS-pDR1M slab. During propagation, the Gaussian beam diameter varies as $w\left( z \right) = w_0 \times \sqrt {1 + \left( {\frac{{\lambda _wz}}{{n_0\pi w_0^2}}} \right)^2}$ and the intensity drops off as *e*^−*αz*^ at *λ*_*w*_ because of the absorption by the azo-groups. An absorption coefficient of *α* = 0.379 mm^−1^ is estimated from longitudinal fluorescence measurements, as detailed in Supporting Information.

The maximum value for the light-induced refractive index $n_{{\rm ind}}^{\max }$ can be deduced by comparing the measured phase values (baseline subtracted) corresponding to the maximum intensity of the ‘writing’ beam (at *x* =*y* =0) and outside the region irradiated by the ‘writing’ beam. Since the phase accumulated by the ‘probe’ beam while propagating through the slab results from an integration along the optical path, the phase difference can be calculated as follows:

3 $$\left| {\Delta \Phi _{\max }} \right| = \left| {\Phi _{00} - \Phi _{{\rm outside}}} \right| = \frac{{2\pi }}{{\lambda _p}} \cdot \left| {{\int}_0^h {n\left( {0,0,z} \right){\rm d}z - n_0h} } \right|\\ = \frac{{2\pi }}{{\lambda _p}} \cdot \left| {{\int}_0^h {n_{{\rm ind}}\left( {0,0,z} \right){\rm d}z} } \right| = \left| {\Phi _{00}} \right|$$

where $\Phi _{00}$ is the phase (baseline subtracted) retrieved on the optical axis, $\Phi _{{\rm outside}}$ is a constant phase value associated with points outside the region irradiated by the ‘writing’ beam, $\left| {\Delta \Phi _{\max }} \right| \approx 30\pi$ is the maximum phase difference extracted from Fig. 3b, h is the total thickness of the PDMS-pDR1M slab. Equation (3) can then be solved numerically, resulting in $n_{{\rm ind}}^{\max } = 5.6 \times 10^{ - 3}$.

**Fig. 4:**
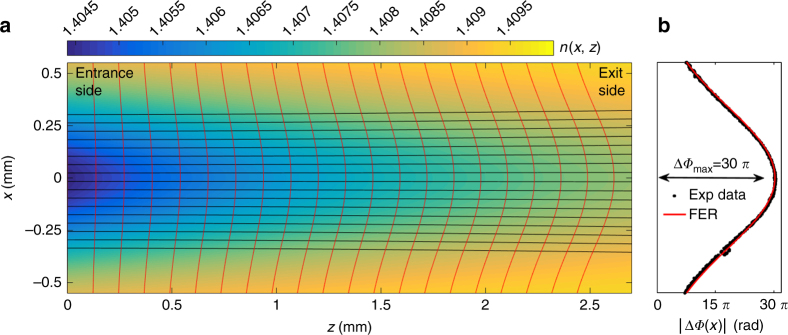
Wavefront deformation through illuminated PDMS-pDR1M slab. **a** Cross-sectional 2D representation of the PDMS-pDR1M slab on the (*x*,*z*) plane showing the GRIN distribution (false color map), the corresponding ray trajectories calculated by FER (black solid lines) and several phase profiles accumulated during propagation (red solid lines, not to scale). **b** Calculated Gaussian phase profile of the ‘probe’ beam at the exit facet of the slab superposed onto the measured phase profile (from Fig. 3b)

The FER model can be used to compute the trajectories of rays transmitted through a PDMS-pDR1M slab characterized by the GRIN distribution *n*(*x, y, z*) calculated above. However, due to rotational symmetry of the 3D distribution *n*(*x,y,z*), a simpler 2D representation can be used instead, such as $n\left( {x,y = 0,z} \right) = n\left( {x,z} \right)$. Figure 4a shows the GRIN function $n\left( {x,z} \right) = n_0 - n_{{\rm ind}}^{\max } \times ( {\frac{{w_0}}{{w\left( z \right)}}} )^2 \times e^{ - \frac{{2x^2}}{{w\left( z \right)^2}}} \times e^{ - \alpha z}$ defined over a 2D longitudinal slice of the PDMS-pDR1M slab and the corresponding calculated ray trajectories. Since the ‘probe’ beam is well-collimated and aligned along the optical axis, all incoming rays are incident parallel to the optical axis, regardless of their incidence position *x* along the slab entrance facet (plane-wave approximation). According to Huygen’s principle, the exit angles θ_out_(*x,z* = *h*) for rays leaving the GRIN domain are related to the overall wavefront shape of the transmitted light. To appreciate the deformation of the initially flat wavefront, the function $\frac{{2\pi }}{{\lambda _p}}n\left( {x,z} \right)$ is integrated along each ray trajectory *L*_*i*_ (i=1, …, *N*), where *N* is the total number of rays considered. As a result, a Gaussian phase profile is obtained, as shown in Fig. 4b, which thus validates the use of the FER model for the axis-symmetric light-induced GRIN distribution. For the case of the integration being limited to progressively increasing portions of the ray trajectories $L_i$, it is possible to appreciate the evolution of the wavefront during the beam propagation (red lines in Fig. 4a).

**Fig. 5:**
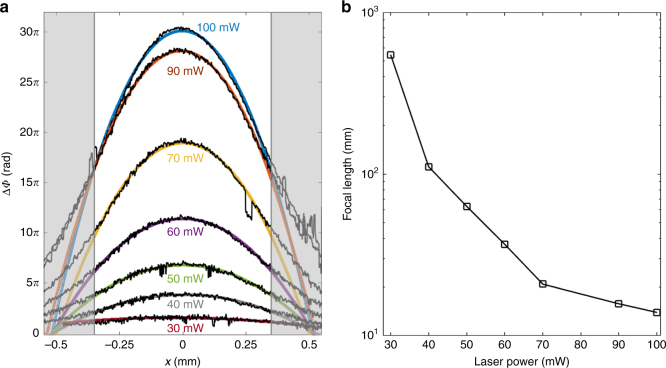
Intensity-dependent photoinduced GRIN distribution. **a** Phase profiles (black solid lines) of the ‘probe’ beam for different intensities of the ‘writing’ beam and corresponding parabolic fits, as indicated (colored solid lines). **b** Focal lengths of the PDMS-pDR1M as a function of the ‘writing’ beam power

The maximum value of the light-induced refractive index distribution $n_{{\rm ind}}^{\max }$ can be varied by varying the ‘writing’ beam power. In Fig. 5a, several phase cross-sections measured for different writing power conditions are shown, wherein the maximum phase difference increases with increasing writing power. When the Gaussian ‘writing’ beam power is below 100 mW (while keeping the beam waist constant at *w*_0_=650 μm), the retrieved phase variation $\left| {\Delta \Phi _{\max }} \right|$ and, hence, the maximum photo-induced refractive index change $n_{{\rm ind}}^{\max }$ scale linearly with the laser power. However, at higher irradiation energy densities (i.e., tighter focusing or higher power of the ‘writing’ beam), $n_{{\rm ind}}$can depart from the Gaussian shape and eventually exhibit a nonlinear response^37^. As a result, the measured phase fails in providing reliable information for the actual distribution of the refraction index in the slab volume (see Supporting Information). Within an inner 700-μm-wide region, a parabolic profile $\Delta \Phi \left( x \right) = ax^2 + bx + c$ is well fitted to the measured phase for the entire range of writing power considered. In the Fresnel approximation, the transmission function of a thin lens along one direction has a parabolic phase $\propto \exp ( { - \frac{{2\pi }}{{\lambda _{}}}\frac{{x^2}}{{2f}}} )$, where *f* is the lens focal length and *x* is a transverse axis on the lens plane. From the coefficients of the parabolic fit, it is straightforward to calculate the equivalent focal length of the PDMS-pDR1M GRIN slab as $f = \pi \lambda _p^{ - 1}a$ at different ‘writing’ beam power. The results of such a calculation are shown in Fig. 5b, demonstrating focal lengths as low as 14 mm (corresponding to an NA = 0.025 for a 700 μm-wide entrance pupil) at maximum writing power.

**Fig. 6:**
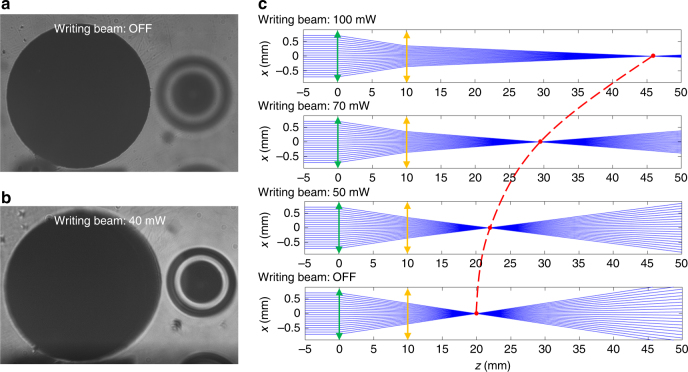
Tunable-focus microscope. **a** White-light image of the top pattern of the sample in a switched-off ‘writing’ beam state. **b** White-light image of the bottom pattern of the sample in a switched-on ‘writing’ beam state (~40 mW). The two patterns are longitudinally separated by a 1-mm-thick glass slide. See Movie M1 for real-time operation. **c** Ray tracing simulation of an imaging system composed of an objective (focal length 20 mm) placed at *z *= 0 mm (green double arrow) and the PDMS-pDR1M GRIN element placed at *z* = 10 mm (yellow double arrow). The focal lengths of the PDMS-pDR1M element at different ‘writing’ beam intensities are obtained from Fig. 5b

The tunable-focus capability of the PDMS-pDR1M element is exploited for white-light imaging, using the setup described in the Materials and method section, wherein the ‘probe’ beam is replaced by a halogen lamp and the reference beam is blocked. The PDMS-pDR1M slab is placed in between the collection objective and the sample, at a distance of 10 mm from the objective. The sample object is a glass slide that is 1 mm thick, with two chromium patterns lithographed on each side and positioned in such a way that the top pattern is imaged onto the CMOS camera (Fig. 6a). When the ‘writing’ beam is switched on, a progressive increase of the effective focal length of the system is produced, until the bottom pattern can be imaged through the glass slide (Fig. 6b). The continuous change in focal length with time can be appreciated by watching Movie M1.

Ray tracing code based on the ray transfer matrix^38^ can be used to assist in predicting the position of the focal plane for the complete system comprising the objective (NA = 0.42, focal length of 20 mm) and GRIN element under different ‘writing’ beam power conditions (Fig. 6c). Since the lateral size of the GRIN element useful for imaging is limited to 700 μm (the linear range of the parabolic fit in Figs. 2 and 4), only a 1.4-mm-wide central region of the objective entrance pupil is considered in the calculations. When the ‘writing’ beam is switched off, the focal plane is at a distance of 20 mm from the objective (i.e., the focal distance of the collection objective). By increasing the ‘writing’ beam power, the focal plane position increases until a 45 mm focus is reached at the maximum power. Despite a general decrease of the overall numerical aperture due to the tiny size of the GRIN element, the adjustable-focus capabilities of the imaging system are thus demonstrated.

**Fig. 7:**
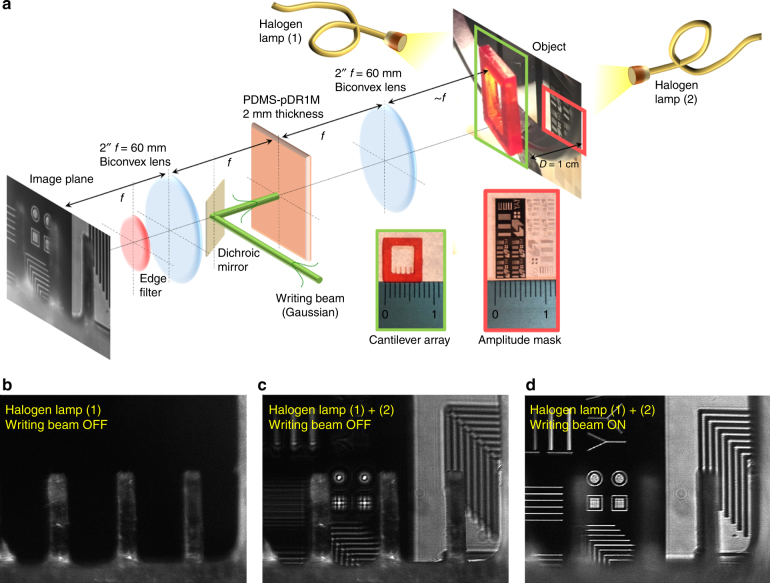
Low-magnification, white-light imaging system. **a** Schematic of a 4-f imaging system based on 2″ biconvex lenses. Imaged objects are represented by an amplitude mask and a 3D-printed cantilever array (see insets) that are axially separated by an air gap that is ~1 cm wide. The PDMS-pDR1M slab is placed in the pupil-conjugated plane of the first collection lens. Illumination is provided by two separate halogen lamps enabling the objects to be imaged upon collection of both scattered and transmitted light. **b** Illumination by the halogen lamp (1): the cantilever array is imaged in-focus (scattered light). **c** Illumination by both halogen lamps (1) and (2): the cantilever array is imaged in-focus (scattered light) and the amplitude mask is out-of-focus (transmitted light). **d** Illumination by both halogen lamps (1) and (2): the cantilever array is out-of-focus (scattered light) and the amplitude mask is in-focus (transmitted light) following irradiation of the PDMS-pDR1M slab by the ‘writing’ beam. See Movie M2 for operation

As an alternative application, a white-light imaging system based on a pair of 2-inch biconvex lenses (focal length of 60 mm) is proposed (Fig. 7a), wherein the PDMS-pDR1M slab is positioned in the pupil-conjugated plane of a first collection lens, as commonly utilized in microscopy systems employing tunable lenses^39,40^. The lens configuration is 4-f; therefore, the overall magnification is close to unity. A dichroic mirror is positioned in between the two lenses such that the *λ*_*w*_ = 532 nm ‘writing’ beam is reflected toward the PDMS-pDR1M slab, while red-IR radiation is transmitted along the optical axis. An edge filter is placed in front of the CMOS camera, which blocks stray laser light and residual reflections from the dichroic mirror. The imaged objects are composed of a scattering element (a polymeric 3D-printed cantilever array) and an amplitude mask (a photolithographic plate), which are axially separated by a gap of *D* = 1 cm. Two independent halogen lamps are used to illuminate the objects from the rear of the amplitude mask and from the front of the cantilever array. Due to the large size of the optical elements used, the field of view of this system is ~5 mm. The objects are placed such that the cantilever is normally in-focus when illuminated by the halogen lamp (1), as shown in Fig. 7b. In fact, when the halogen lamp (2) is switched on, the amplitude mask looks out-of-focus, while the cantilevers are still in-focus (Fig. 7c). As the writing beam (Gaussian shape) is illuminating the PDMS-pDR1M in the back focal plane, an approximately parabolic phase profile is produced, which brings the amplitude mask in-focus onto the image plane (Fig. 7d). In movie M2, the operation of the 4-f system can be appreciated.

We observe here that the advantage of inserting the PDMS-pDR1M slab into the back focal plane of the first collection lens relies on the opportunity to limit the light-induced refractive index change over a small region of interest, thus alleviating the need for high-power laser sources. In addition, this choice opens the way for further miniaturization and integration of the polymeric tunable GRIN element in imaging systems.

## Conclusions

A light-responsive elastomeric material is introduced whose transmission function can be reversibly engineered by laser irradiation. Unlike most state-of-the-art tunable optical devices, for which different optical functionalities are obtained by pre-patterning or pixelization of the device, a continuous GRIN distribution can be induced here by simple projection of a desired pattern, similar to optically addressed spatial light modulators. However, the resulting transmission function is phase-only, polarization insensitive, and broadband (outside the absorption band of the azo-groups), leading to a much wider range of opportunities for applications. Reversibility (due to use of the elastomeric matrix) enables a full recovery of the refractive index distribution within a few seconds. As illustrative examples, lens like, and axicon-like transmission functions were demonstrated in this work.

GRIN elements are very useful for the elimination of spherical aberrations typical of standard optics^41^, with the flatness of their surface facilitating integration into complex optical microsystems^42^. The results presented here pave the way toward optically driven adaptive GRIN elements that can be used for dynamic correction of focal length or aberrations in imaging systems, as well as in applications requiring dynamically adjustable wavefront engineering.

## Electronic supplementary material

Supplemental material

White Light Microscopy Imaging System

White light imaging system with 2-inch optics

Axicon-like operation
