# Supplementary material for: Reconfigurable elastomeric graded-index optical elements controlled by light
Source: Light Sci Appl. 2018 May 23;7:7. doi: 10.1038/s41377-018-0005-1 (PMC6106995; doi:10.1038/s41377-018-0005-1)
Supplement: Supplementary file 1 — Supplemental material [file 41377_2018_5_MOESM1_ESM.docx]

Supplementary Information

Reconfigurable elastomeric graded-index optical elements controlled by light

*Angelo Angelini*, Federica Pirani, Francesca Frascella, and Emiliano Descrovi**

^1^Department of Applied Science and Technology (DISAT), Politecnico di Torino, C.so Duca degli Abruzzi 24, Torino, IT-10129, Italy.

^2^Center for Sustainable Future Technologies@PoliTo, Istituto Italiano di Tecnologia, C.so Trento 21, Torino, IT-10129, Italy.

**Sample preparation**

In sample preparation, the choice of the azo-polymer and the PDMS compound is crucial to obtain good optical quality. In Figure S1 three different sample preparations are shown.


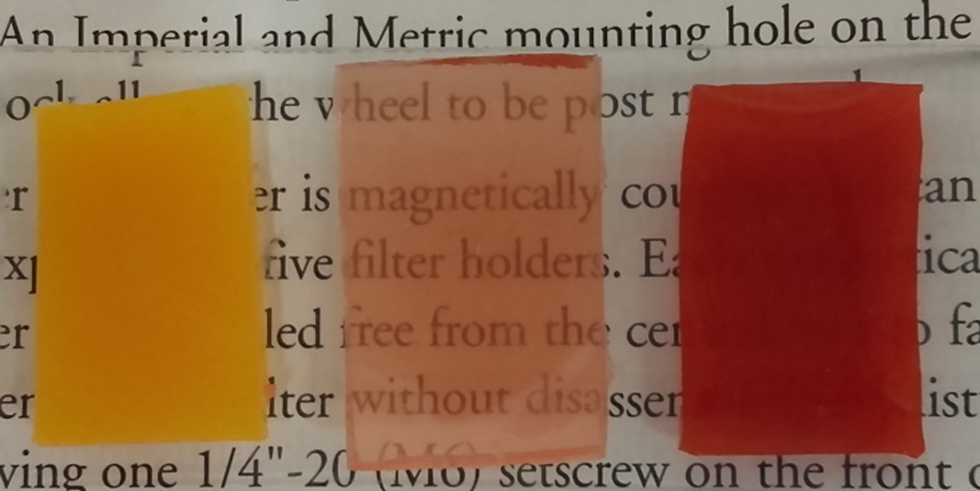


**Figure S1.** PDMS slabs embedding different azopolymers: PAZO (left), DR1 (center and right).

On the left side (yellow sample), the compound is a mixture of poly[1-[4-(3-carboxy-4-hydroxyphenyl-azo) benzene sulfonamido]-1,2- ethanediyl, sodium salt] (PAZO), a commercial azo-polyelectrolyte well known for its high photo-responsivity. The sample appears as rather opaque because of the high scattering effects, raising from a bad dissolution of PAZO in PDMS.

Samples in the middle and on the right are obtained by mixing another commercial azo-polymer (Dispersed Red 1 -DR1-) with PDMS at two different concentrations (70 µl and 300 µl per ml of PDMS, respectively). As in the previous case, the azo-polymer is badly dissolved, resulting in scattering effects that are increasing at increasing concentrations of DR1. Instead, the PDMS-pDR1M sample described in the main text and shown in Figure 1 shows good optical quality also at high concentration, thanks to the good miscibility of poly-Dispersed Red 1 Methacrylate into PDMS.

**Estimation of the absorption coefficient**

The parameter $\alpha$*α* employed in the FER model represents the absorption coefficient of PDMS-pDR1M at *λ_w_*=532 nm. A direct estimate of the absorption coefficient can be performed by imaging the longitudinal fluorescence distribution emitted by the sample when excited by the ‘writing’ beam (Figure S2a). To this aim, the illumination direction is rotated by 90 degrees, in such a way that the ‘writing’ beam propagates transversely to the optical axis of the imaging system (see inset in Figure S2a).


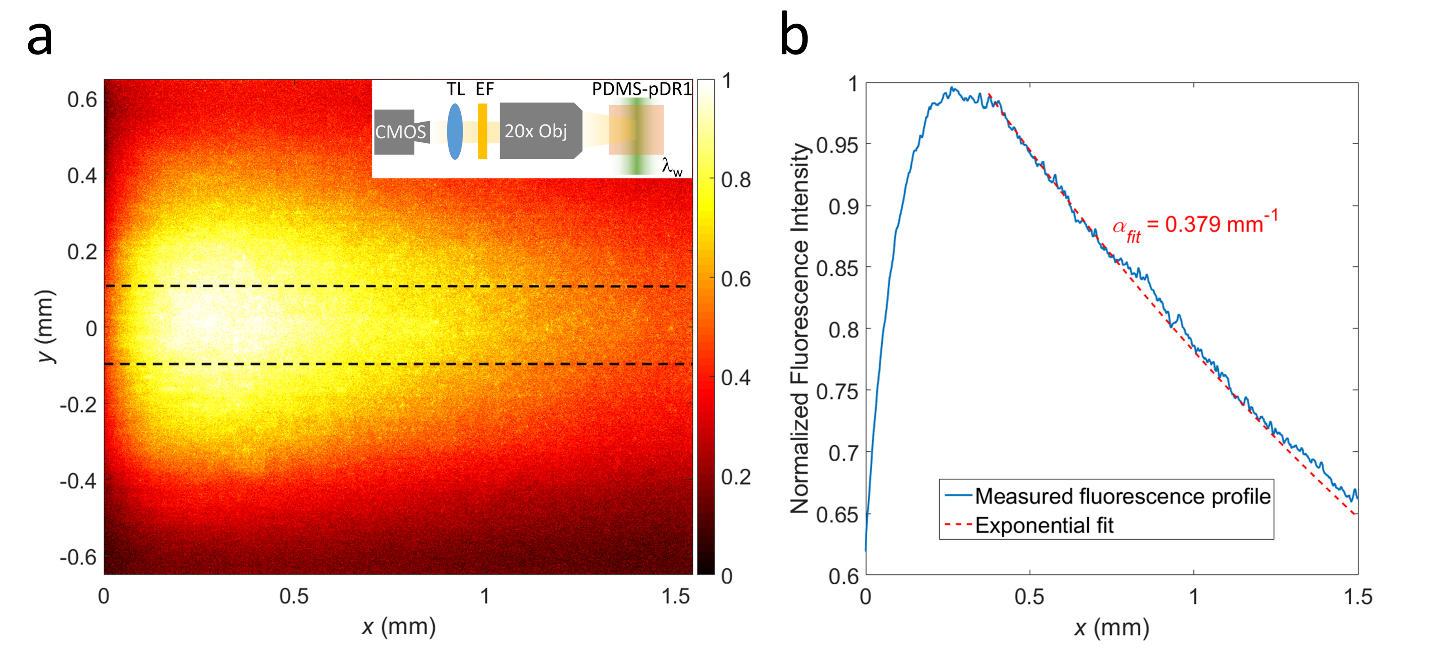


**Figure S2.** a) False-color representation of the longitudinal distribution of fluorescence excited by the ‘writing’ beam (see inset for the geometrical configuration). Fluorescence is distributed according to the Gaussian ‘writing’ beam that is exponentially decay along the propagation direction. b) longitudinal cross-section of measured fluorescence intensity revealing the single-exponential decay with slope *α_fit_*=0.379 mm^-1^.

As evident from Figure S2a, the exponential decrease of the fluorescence emission follows a corresponding decrease of the ‘writing’ beam intensity along the propagation direction. A representative fluorescence decaying profile (Figure S2b) is obtained by averaging the collected intensity from a transverse region defined by the two black dashed lines in Figure 2Sa. Apart from an initial region where diffraction effects from the slab edge dominate, the fluorescence profile can be well fitted by a single-exponential function. The resulting slope is given by the absorption coefficient *α*=0.379 mm^-1^.

**Polarization sensitivity**

The polarization sensitivity of the light-induced refractive index change effect in the PDMS-pDR1M slab is evaluated by measuring the phase distribution of the ‘probe’ beam in two orthogonal polarization states. In Figure S3a,b the baseline-subtracted unwrapped phase distributions as measured by a ‘probe’ beam linearly polarized either parallel or orthogonal to the ‘writing’ beam are presented. Apart from a slight spatial shift due to a refraction effect due to the Half Wave Plate used to rotate the ‘probe’ beam polarization, the two phase distributions look very similar. More specifically, the cross-sectional phase profiles taken along the dashed lines in Figure S3a,b can be almost completely overlapped, thus demonstrating the basically isotropic refractive index changes induced by a linearly polarized ‘writing’ beam.


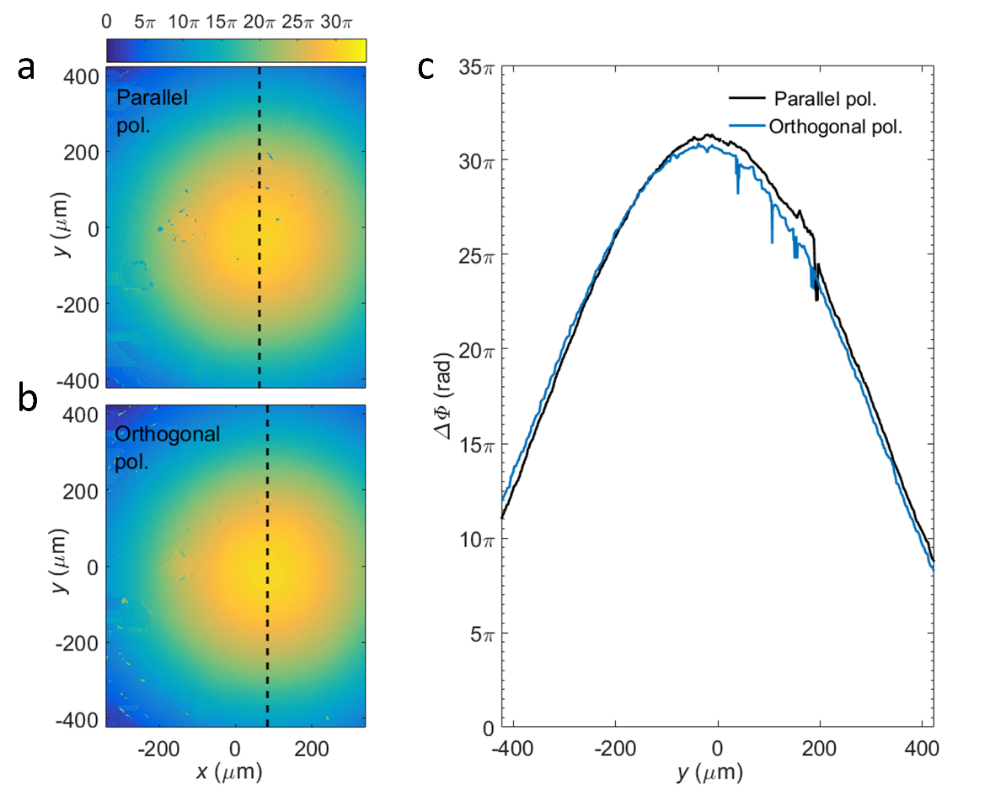


**Figure S3.** Baseline-subtracted unwrapped phase distributions of the ‘probe’ beam with polarization parallel (a) and orthogonal (b) to the ‘writing’ beam. c) Cross-sectional phase profiles along the y-cuts indicated as dashed lines in (a) and (b). ‘Writing’ beam waist is *w_0_*≈650 μm and beam power is 100 mW.

**Temporal Dynamics**

The dynamics of the light-induced refractive index change is investigated by considering the time evolution of the interference fringes produced in the image plane of the optical setup shown in Figure 2 as the PDMS-pDR1M slab is irradiated by the ‘writing’ beam. To this end, the CMOS camera is replaced by a photodiode positioned at the center of the ‘writing’ beam as imaged on the image plane. When the ‘writing’ laser is switched on at *t=*0, the photodiode signal acquisition is started and a temporal trace is then recorded. As soon as a refractive index change in the PDMS-pDR1M slab is triggered, running interference fringes are continuously produced in the interferometer image plane, thus time-modulating the light intensity detected by the photodiode. When a stationary condition is reached, no new fringes are produced and the light intensity detected by the photodiode reaches a constant value. The cumulative number of intensity modulations detected at a given instant *t* after the process has been triggered indicates the overall phase shift $\Delta\Phi\left(t\right)$ induced at the PDMS-pDR1M point that is imaged onto the photodiode. As a result, a dynamic curve $\Delta\Phi\left(t\right)$ can be derived, as shown in Figure S4 for several values of the ‘writing’ beam power.


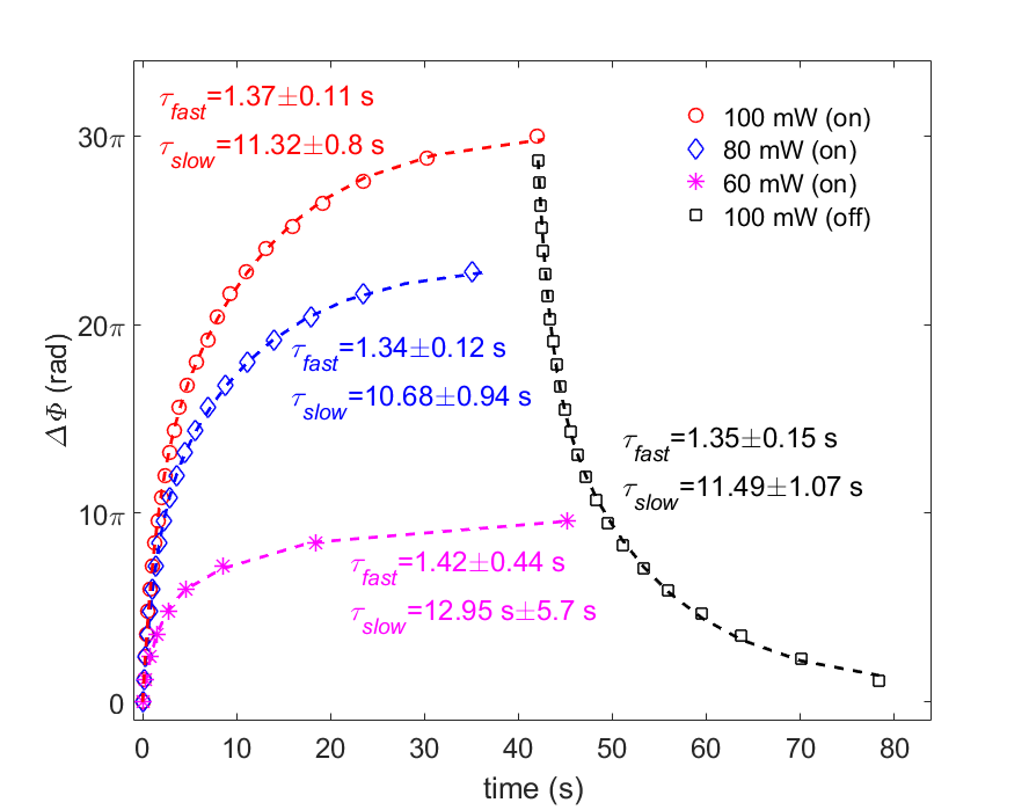


**Figure S4.** Temporal evolution of the phase $\Delta\Phi\left(t\right)$ induced in the PDMS-pDR1M slab by a 650 μm waist ‘writing’ beam having different power values: 100 mW (red circles), 80 mW (blue diamonds), 60 mW (magenta stars). The relaxation dynamic starting when the ‘writing’ laser (power 100 mW) is switched off is also shown (black squares). Experimental data is well-fitted by a weighted sum of two exponential functions having a fast and a slow time constant *τ_fast_* and *τ_slow_* respectively. Fitting curves are plotted as dashed lines.

Experimental data reported in Figure S4 can be well-fitted by a weighted sum of two exponential functions characterized by a fast and a slow time constant τ_fast_ and τ_slow_ respectively. When the ‘writing’ beam is switched off, the PDMS-pDR1M starts to relax back to the initial unperturbed state ($\Delta\Phi\left(0\right)$=0) following a similar temporal dynamics. The time constants τ_fast_ and τ_slow_ differ by roughly one order of magnitude, thus suggesting that the refractive index change induced by the ‘writing’ beam rely on two different physical effects. We speculate that the fast refractive index change may be due to the continuous photo-isomerization of the pDR1M azo-groups, while the slowly-varying component may be due to a thermal expansion due to laser heating.

**Conformality of the photo-induced refractive index to the writing beam intensity**

The maximum phase shift induced within the PDMS-pDR1M slab generally scales linearly with the ‘writing’ beam power below 100 mW, provided that the laser energy is confined within a beam radius of about 650 μm at least. This effect is illustrated in Figure S5a, where measured$\Delta\Phi_{\max}$ values are plotted for different power values of two ‘writing’ beams having waist $w_{0}=650$μm and $w_{0}=400$μm respectively. For a beam waist $w_{0}=650$μm and a laser power smaller than 100 mW, the phase profile $\Delta\Phi\left(x\right)$of the ‘probe’ beam and hence the spatial distribution of the induced $n_{ind}$are generally conformal to the ‘writing’ beam intensity. However, if the ‘writing’ beam is scaled down by tight focusing, the emerging phase of the ‘probe’ beam departs from the Gaussian shape of the ‘writing’ beam, even at a relatively moderate power (Figure S5b). This effect results from refraction occurring within the GRIN medium due to the strong gradient of the induced refractive index $n_{ind}$. When the laser power is increased, the induced $n_{ind}$may exhibit an additional non-linear dependence on the ‘writing’ beam intensity, as expected in azopolymeric compounds. Because of the combination of these two effects, the phase measurement fails in providing reliable information on the actual spatial distribution of the refraction index in the slab volume.


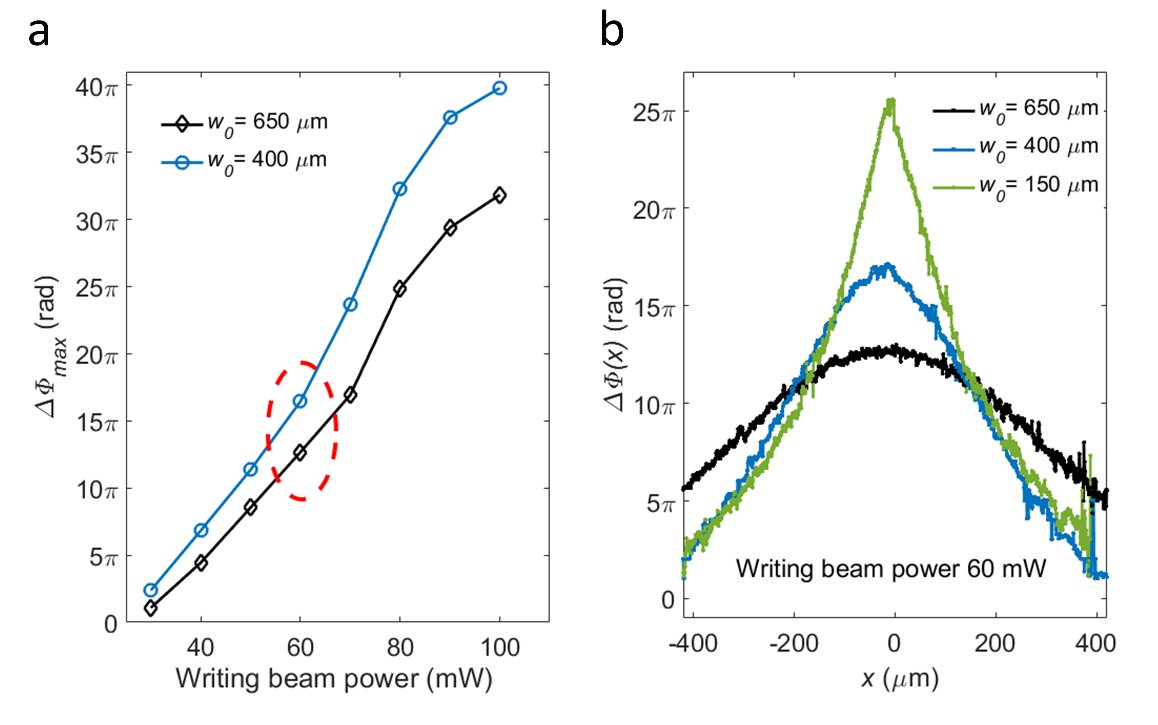


**Figure S5.** a) Maximum measured phase shift of the ‘probe’ beam transmitted through a PDMS-pDR1M slab (2.7 mm thickness) during a simultaneous irradiation by a Gaussian ‘writing’ beam at increasing laser power and different waist values. b) Exemplary phase profiles $\Delta\Phi\left(x\right)$ obtained upon irradiation of a Gaussian ‘writing’ beam at 60 mW power, focused down to waist $w_{0}=650$μm (black curve), $w_{0}=400$μm (light blue curve), $w_{0}=150$μm (light green curve). Deviations of the retrieved phase profiles from a Gaussian shape can be appreciated for smaller beam waists.

**Axicon-Like GRIN**

The FER model introduced above is useful to predict the optical response of a GRIN element in case of different ‘writing’ intensity distributions. In the following, the effect of the ‘writing’ beam focusing is investigated by considering ‘writing’ beams incident on the entrance facet of the PDMS-pDR1Ms slab with a smaller waist w_0_, while keeping the maximum intensity constant. Depending on the ‘writing’ beam waist, the light-induced GRIN spatial distribution as well as the light-induced phase function change accordingly. For a Gaussian GRIN distribution having *w_0_*=50 μm and $n_{ind}^{\max}=5.6\cdot10^{-3}$, ray trajectories are calculated (Figure S6a) and the angular coefficients for each individual ray leaving the slab domain, i.e. $tg\theta_{out}\left(x,z=h\right)$, are plotted $\vartheta_{\mathrm{out}}$as a function of the ray exit positions (Figure S6b). Due to the strong refractive index gradient within the slab, light rays are drastically deviated, with a maximum angular spread of$\theta_{out}\cong2^{o}$ with respect to the optical axis. As a result, an inner dark region surrounded by a bright corona is produced. Taking into account the axial symmetry of the 2D slab domain used in the FER calculation, such a light distribution has a quasi-conical shape in 3D, thus suggesting that the GRIN distribution in the PDMS-pDR1M slab operates similarly to a concave axicon. For comparison purposes, the distribution of $tg\theta_{out}\left(x,z=h\right)$ for a Gaussian GRIN distribution having *w_0_*=650 μm and $n_{ind}^{\max}=5.6\cdot10^{-3}$ is also shown in Figure S6b. A linear trend is found in this case, as expected for rays associated to the parabolic wavefront emerging from a low NA lens illuminated by a plane wave.


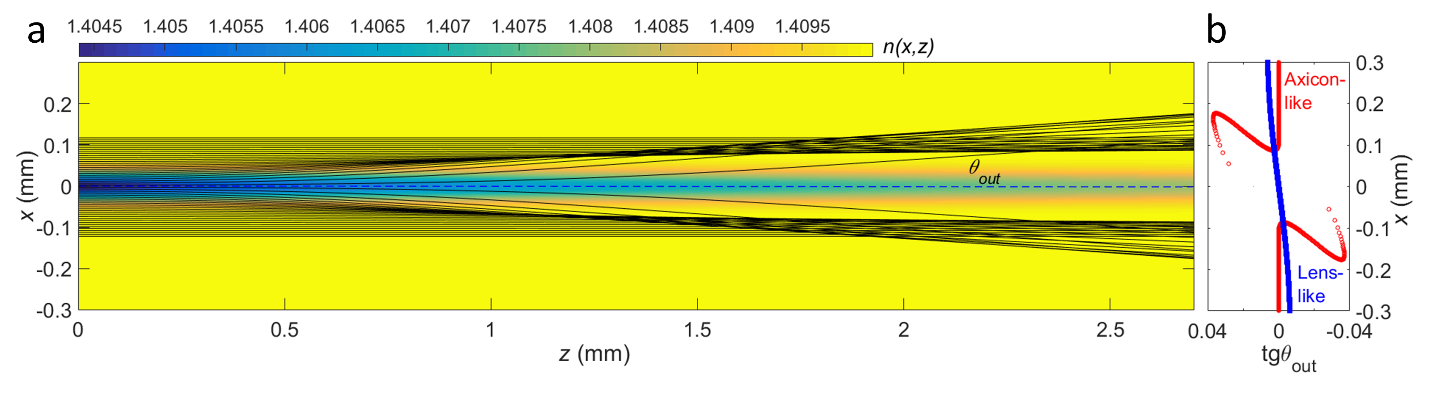


**Figure S6.** a) FER-modeled ray trajectories for a PDMS-pDR1M slab having a Gaussian GRIN distribution with $n_{ind}^{\max}=5.6\cdot10^{-3}$ and *w_0_*=50 μm (false color map). b) Angular coefficient of rays leaving the PDMS-pDR1M slab as a function of their transverse position *x* at the slab exit side, for a w_0_=50 μm (axicon-like, red circles) and a *w_0_*=650 μm (lens-like, blue squares) GRIN distribution

While the distribution $tg\theta_{out}\left(w_{0};x,z=h\right)$ is useful for understanding the wavefront distortion produced by the GRIN distribution, for a predictive analysis it is also important to calculate the exit angle $\theta_{out}$ as a function of the optical ray input position. In Figure S7a, a false color map of the function $tg\theta_{out}\left(w_{0};x_{in}\right)$ is presented, wherein *x_in_* is the entrance transverse position of the input rays. For large ‘writing’ beam waists, the angle$\theta_{out}$ is linearly distributed along the transverse direction *x_in_*. As rays are perpendicular to the local wavefront, a linear distribution of $tg\theta_{out}$ is expected for the parabolic wavefront as emerging from a low NA lens. For smaller ‘writing’ beam waist, $tg\theta_{out}$ reveals that rays are more and more deflected as their entrance position moves apart from the optical axis, until a maximum deflection angle is reached (Figure S7b). Then, as rays fall outside the modulated GRIN region, $tg\theta_{out}$ decreases back to zero. As a result, a range of diverging angles can be produced for a light radiation incident onto the PDMS-pDR1M slab depending on the relative alignment of the ‘writing’ beam axis with respect to the ‘probe’ radiation transmitted though the slab.


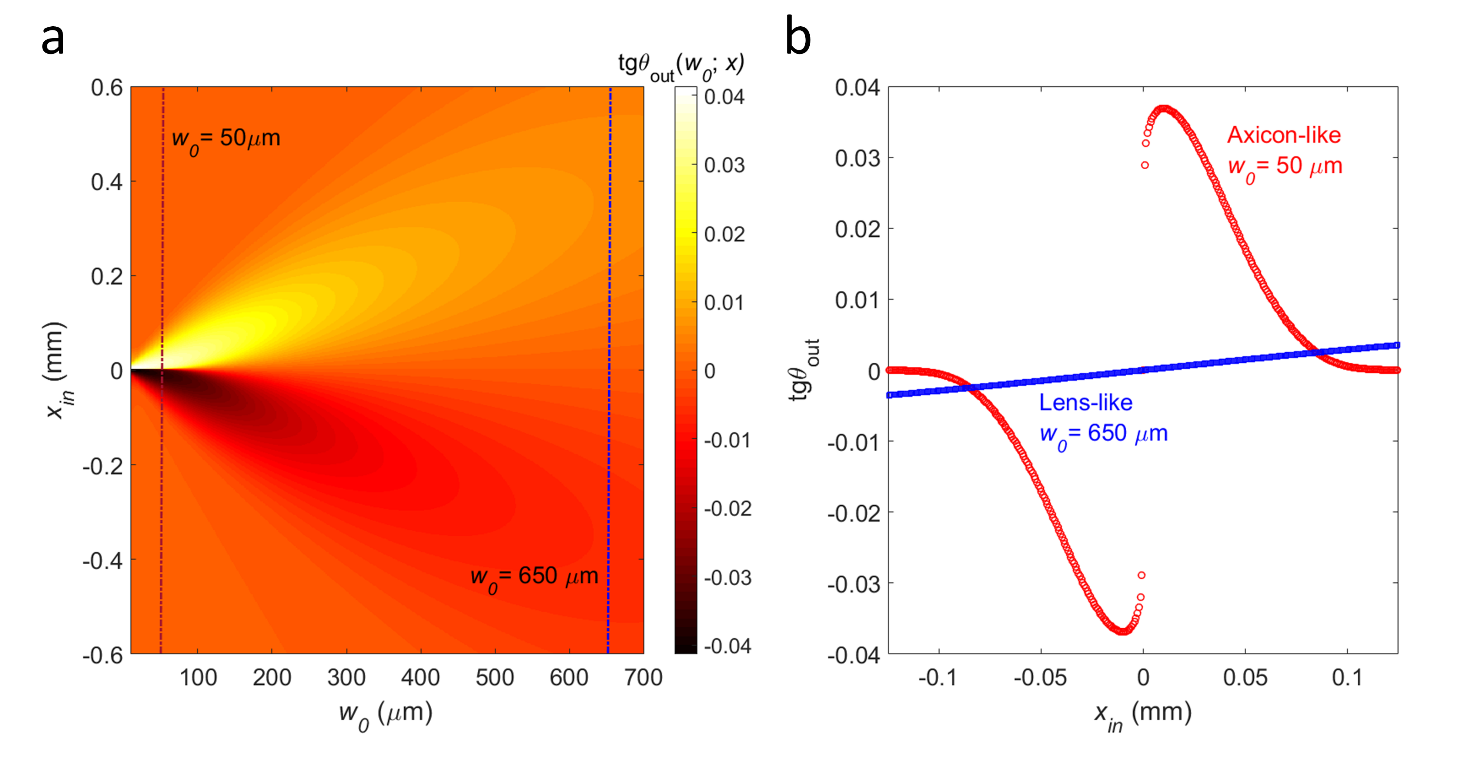


**Figure S7.** a) False-color representation of $tg\theta_{out}\left(w_{0};x_{i}\right)$ for different ‘writing’ beam waist *w_0_*, as a function of the ray entrance positions *x_i_*. b) Cross-sectional profile of $tg\theta_{out}\left(w_{0};x_{in}\right)$ for *w_0_*=50 μm (red circles) and *w_0_*=650 μm (blue squares).

Experimentally, the ‘writing’ beam is focused down to a waist *w_0_*=60 μm $50 \mu m$and the resulting ‘probe’ beam intensity distribution on the top surface of the PDMS-pDR1M slab is imaged at increasingly higher writing power (see Supplementary Movie M3). In particular, the ‘probe’ beam intensity resulting from a 100 mW writing power is shown in Figure S8a. As expected by previous calculations, when the induced GRIN gradient is strong enough, rays are dramatically deviated from the initial propagation direction, resulting in a narrow bright corona surrounding a dark cone in the forward direction, similarly to the case of a concave axicon-like element. Because of such a strong ray deviation, a direct interferometric estimation of the light-induced $n_{ind}^{\max}$ is thus hampered. We want to underline that the observed pattern exhibits an angular divergence that is much higher than predicted by the linear FER model when fed by a purely Gaussian GRIN distribution.

When the collection system is moved in such a way that the object plane is located about 1 mm below the bottom surface of the PDMS-pDR1M slab, the caustic shown in Figure S8b is imaged. This intensity pattern recalls a Bessel function. However, since the collected light is diverging, the Bessel distribution is virtual. Interestingly, such a pattern is very slowly diffracting and maintains a transverse shape for a millimetric distance far away from the slab.


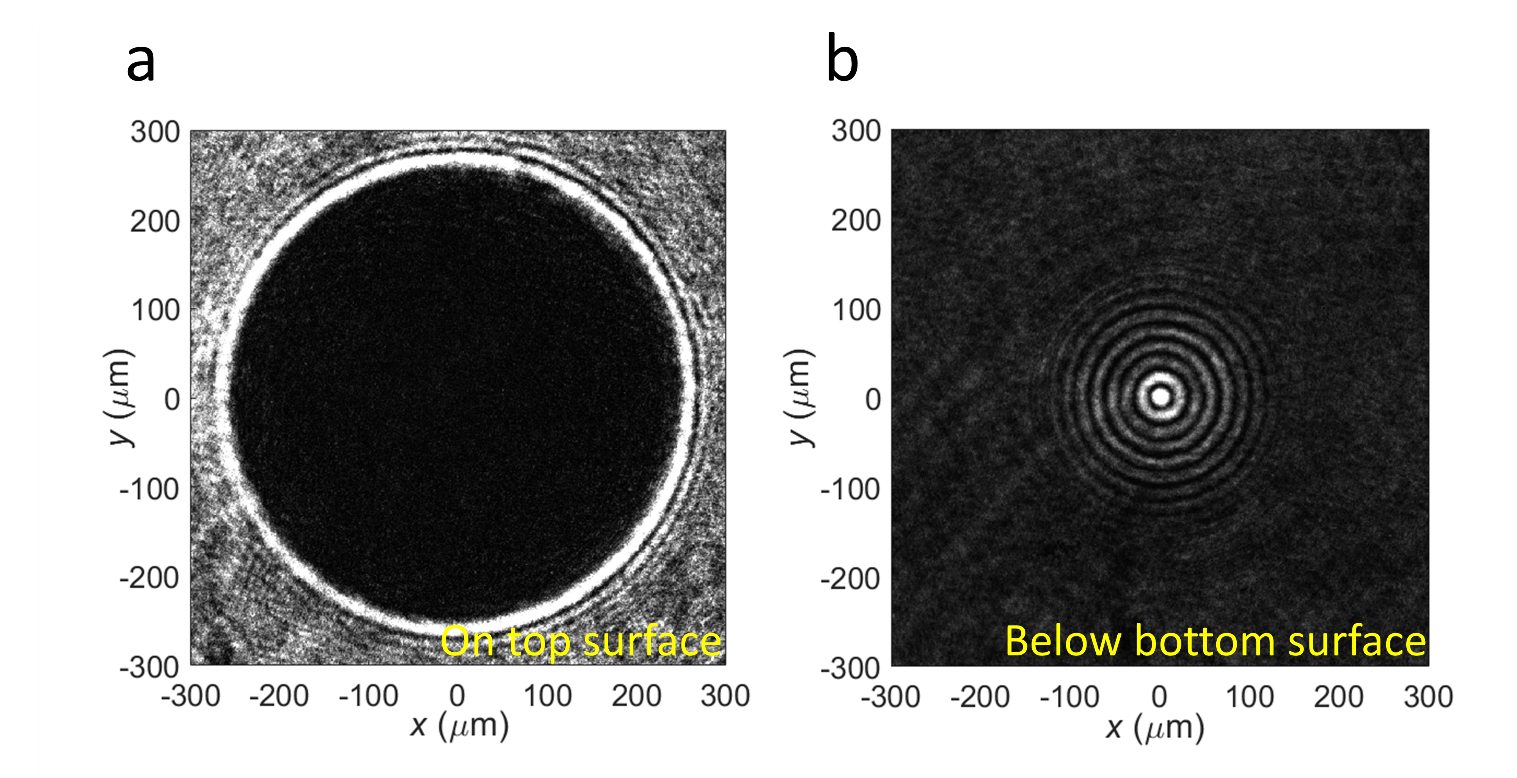


**Figure S8.** a) Image of the ‘probe’ beam intensity at the top surface (exit side) of the PDMS-pDR1M slab irradiated with a *w_0_*=60 mm ‘writing’ beam. A dark cone surrounded by a bright corona is produced as a result of the light refraction due to the strong GRIN gradient induced in the slab. b) Image of the ‘probe’ beam intensity below the bottom surface (illumination side) of the PDMS-pDR1M slab irradiated with a *w_0_*=60 mm ‘writing’ beam. A Bessel-like pattern is imaged as a caustic virtually obtained by back-propagating light emerging from the slab. ‘Writing’ beam power is 100 mW.

**Movie M1**. White light imaging of two lithographed patterns separated by a 1 mm thick glass slide. The continuous change in focal length is controlled by the ‘writing’ beam (spectrally filtered before reaching the CMOS camera) incident on the PDMS-pDR1M slab at increasing power.

**Movie M2**. White light imaging of an amplitude mask and a polymeric cantilever array axially separated by an air gap about 1 cm wide. The system is configured as a 4-f imaging system employing 2” biconvex lenses having 60 mm focal length. The PDMS-pDR1M element is placed in the aperture-conjugated plane of the first collection lens. Illumination is provided by two separate halogen lamps allowing the objects to be imaged upon collection of both scattered and transmitted light. The continuous change in focal length is controlled by the ‘writing’ beam (spectrally filtered before reaching the CMOS camera) incident on the PDMS-pDR1M slab at increasing power.

**Movie M3**. Image of the ‘probe’ beam intensity distribution at the exit facet of the PDMS-pDR1M slab during irradiation by a focused ‘writing’ beam (waist *w_0_*=120 μm) at increasing power.
